# Targeting mitochondrial autophagy for anti-aging

**DOI:** 10.1038/s41420-025-02913-y

**Published:** 2025-12-24

**Authors:** Wenjun Shan, Yuling Liu, Ruying Tang, Hui Li, Hongjun Yang, Longfei Lin

**Affiliations:** 1 Institute of Chinese Materia Medica, China Academy of Chinese Medical Sciences, Beijing, China; 2 Institute of Traditional Chinese Medicine Health Industry, China Academy of Chinese Medical Sciences, Nanchang, China; 3 China Academy of Chinese Medical Sciences, Beijing, China

**Keywords:** Autophagy, Drug development

## Abstract

Mitochondrial dysfunction is one of the core drivers of aging. It is manifested by reactive oxygen species (ROS) accumulation, mitochondrial DNA (mtDNA) mutations, imbalanced energy metabolism, and abnormal biosynthesis. Mitochondrial autophagy maintains cellular homeostasis by selectively removing damaged mitochondria through mechanisms including the ubiquitin-dependent pathway (PINK1/Parkin pathway) and the ubiquitin-independent pathway (mediated by receptors such as BNIP3/FUNDC1). During aging, the decrease in mitochondrial autophagy efficiency leads to the accumulation of damaged mitochondria, forming a cycle of mitochondrial damage-ROS-aging damage and aggravating aging-related diseases such as neurodegenerative diseases and cardiovascular pathologies. The targeted regulation of mitochondrial autophagy (drug modulation and exercise intervention) can restore mitochondrial function and slow aging. However, autophagy has a double-edged sword effect; moderate activation is anti-aging, but excessive activation or dysfunction accelerates the pathological process. Therefore, targeting mitochondrial autophagy may be an effective anti-aging technique; however, future focus should be on the tissue-specific regulatory threshold and the dynamic balance mechanism to achieve precise intervention.

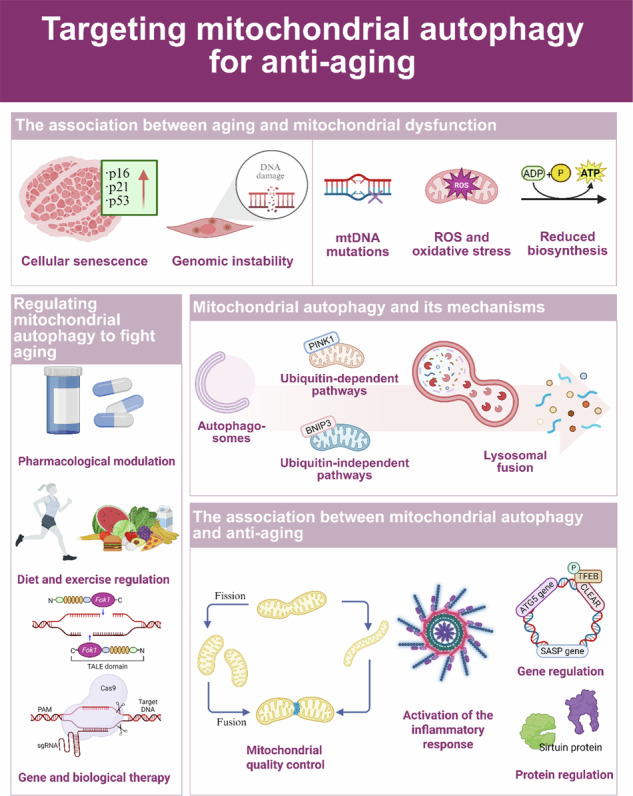

## Facts

- Mitochondrial autophagy acts as both a shield and a spear in aging.
- The balance between AMPK and mTOR is critical for mitochondrial autophagy and SASP.
- NAD+ benefits mitochondrial autophagy, but prolonged elevation may disrupt mitochondrial homeostasis.
- Mitochondrial gene editing in vivo requires further validation before clinical translation.

## Introduction

Cellular aging involves a multilevel functional decline over time, and its pathological basis is closely related to the progressive dysfunction of mitochondria, the center of energy metabolism. As the power plants and signaling hubs of eukaryotic cells, mitochondria maintain cellular homeostasis through core functions such as oxidative phosphorylation (OXPHOS), calcium homeostasis regulation, and apoptosis execution. However, the accumulation of mitochondrial DNA (mtDNA) mutations, reactive oxygen species (ROS) burst, and kinetic imbalance (fusion/disintegration dysregulation) during aging form a cycle, which not only directly impairs ATP synthesis but also drives age-associated pathologies, such as neurodegeneration and cardiovascular diseases, by inhibiting autophagy flow and activating inflammatory pathways. Mitochondrial autophagy is a key quality control mechanism that selectively removes damaged mitochondria and constitutes the core defense against aging. This process is synergistically regulated by two sophisticated pathways: the ubiquitin-dependent pathway in which PINK1 kinase senses the dissipation of mitochondrial membrane potential and recruits the E3 ubiquitin ligase Parkin, which catalyzes the ubiquitin chain labeling of damaged mitochondria, and bridges LC3 molecules via autophagy receptors (OPTN/NDP52) to form autophagosomes; and the ubiquitin-independent pathway in which outer mitochondrial membrane proteins, such as BNIP3 and FUNDC1, anchor autophagy via the LC3 interaction motif (LIR) in response to hypoxia or stress signaling. Notably, autophagy efficiency decreases significantly with aging, leading to the accumulation of dysfunctional mitochondria, which further amplifies oxidative damage and energy crisis, whereas the overactivation of autophagy may lead to excessive mitochondrial clearance and exacerbate metabolic collapse, which is a double-edged sword that highlights the necessity of precise regulation. The current intervention strategies focus on restructuring mitochondrial homeostasis, including (1) pharmacological activation: urolithin A (UA) enhances the PINK1/Parkin pathway, and nicotinamide mononucleotide (NMN) facilitates SIRT3 (a sirtuin)-mediated deacetylation by elevating NAD⁺ levels; (2) gene editing: modulations of Mitochondrial-targeted Transcription Activator-Like Effector Nucleases (mitoTALEN) targets pathogenic mtDNA mutations; and (3) behavioral interventions: caloric restriction activates the AMPK/SIRT1 axis, and exercise training upregulates FUNDC1 expression to restore autophagic activity. This paper systematically analyzes the molecular mechanism, contradictory roles, and targeted intervention strategies of mitochondrial autophagy in aging, aiming to provide a theoretical basis for treatments that delay aging and related diseases.

## The association between aging and mitochondrial dysfunction

### Characteristics of aging and the core mitochondrial role

#### Characteristics of aging

**Fig. 1:**
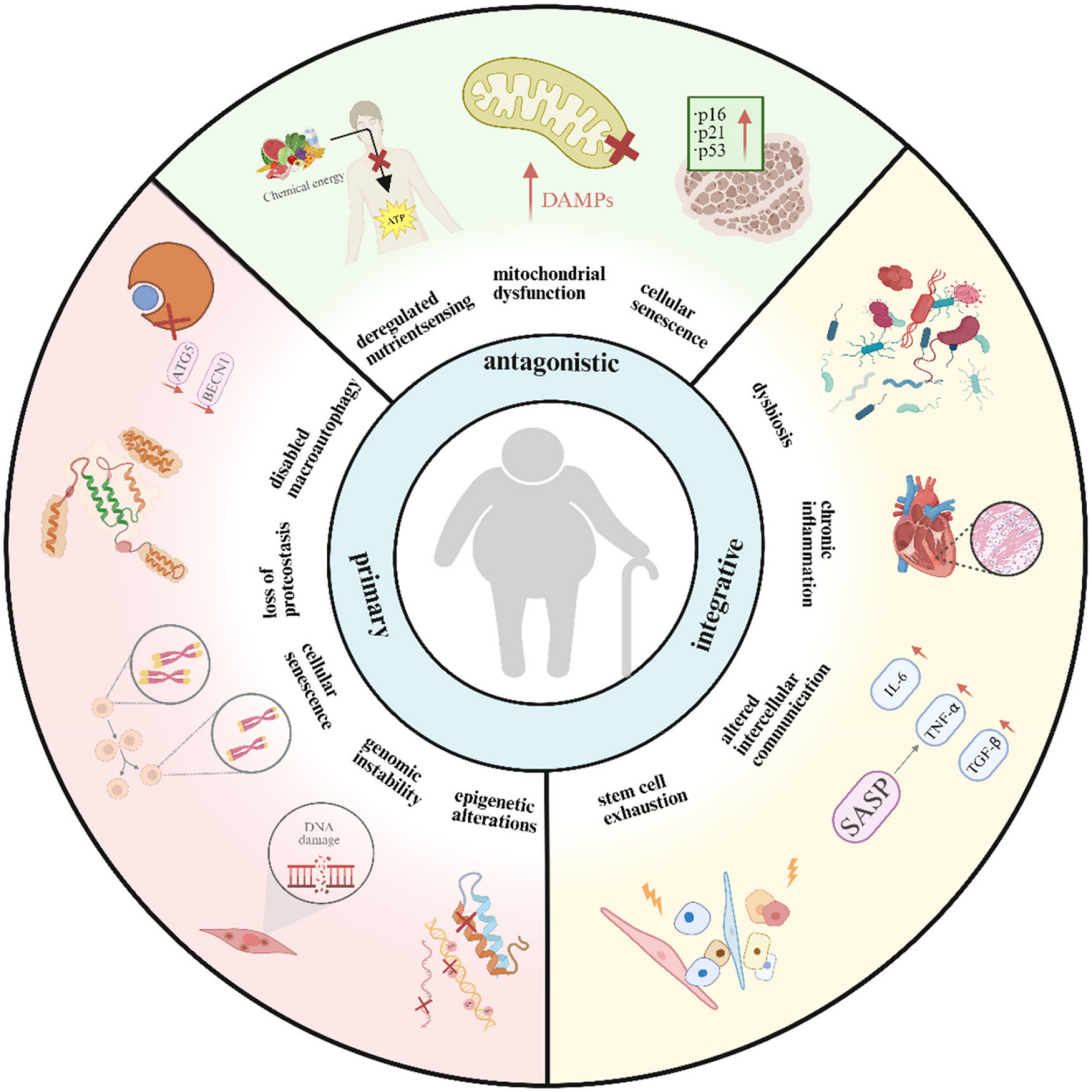
Drivers and features of aging.

The essence of aging is the cumulative multilevel functional decline of an organism over time. Its pathological basis stems from the progressive deterioration of 12 interrelated markers of aging in cells. These hallmarks include (Fig. 1) genomic instability, telomere damage, epigenetic alterations, the loss of protein arrest, the loss of macrophagy, the dysregulation of nutrient perception, mitochondrial dysfunction, cellular senescence, stem cell depletion, altered intercellular communication, chronic inflammation, and ecological dysregulation [1]. Among them, mitochondrial dysfunction and declining autophagy constitute a central hub. The accumulation of mitochondrial DNA mutations and the bursts of ROS in senescent cells further inhibit autophagic flow, leading to the aggregation of aberrant proteins and the accumulation of damaged organelles, and accelerating tissue degeneration [2].

The current study found that aging hallmarks do not exist in isolation but constitute a dynamic network of interactions. For example, sustained DNA damage accelerates telomere depletion, triggering genomic rearrangements and further destabilization, leading to more DNA damage; and the overactivation of mammalian target of rapamycin (mTOR) inhibits autophagy and accelerates mitochondrial function deterioration [3]. Targeted interventions (enhancement of autophagy and epigenetic re-editing) for these features have become key strategies to delay aging and related diseases.

#### Core functions of mitochondria

As the energy metabolism center and multifunctional signaling hub of eukaryotic cells, mitochondria maintain cellular homeostasis through a precisely coordinated biochemical network. Their core functions include: (1) energy conversion: generating ATP through oxidative phosphorylation to drive cellular activities; (2) metabolic integration: regulating the tricarboxylic acid cycle, lipid metabolism, and ketone body synthesis; (3) calcium homeostasis regulation: acting as a calcium reservoir to buffer cytoplasmic calcium ions and regulating endoplasmic reticulum-mitochondria communication; (4) apoptosis execution: releasing cytochrome *c* to activate caspase cascade reactions; and (5) ROS signaling: physiological levels of ROS are involved in regulating cell proliferation and differentiation [4]. During aging, mitochondrial dysfunction is usually manifested as a breakdown of energy metabolism, the dissipation of membrane potential, and the accumulation of oxidative damage [5]. Mitochondrial dysfunction is a result of the synergistic effects of multiple mechanisms, involving structural damage caused by imbalances in mitochondrial dynamics (the downregulation of fusion proteins OPA1/MFN2 and the upregulation of the splitting protein Dynamin-related protein 1); metabolic derangement caused by the compensatory enhancement of glycolysis and insulin resistance induced by lipid accumulation; 10–20-fold increases in the mutation rate of mtDNA with age; genomic destabilization caused by the deletion of key subunits of the complex, for example, MT-ND4 genomic destabilization induced by deletion; and quality control failure triggered by decreased mitochondrial capacity due to defective clearance of the autophagy-lysosome system. These interacting mechanisms ultimately lead to the activation of cellular senescence markers [6, 7]. Therefore, mitochondrial function repair has become an important step in anti-aging interventions.

### Molecular mechanisms associated with mitochondrial dysfunction and aging

#### ROS and oxidative stress

Studies have shown that most intracellular ROS originate mainly from mitochondria, and mitochondrial ROS (mtROS) are generated during OXPHOS that occurs on the mitochondrial electron transport chain (ETC) in the inner mitochondrial membrane [8]. The mitochondrial ETC is a redox hub that regulates cellular homeostasis by producing ATP and ROS. ETC dysfunction, especially decreases in mitochondrial energy production and increases ROS production, is related to the onset and progression of many biological changes leading to obesity and aging, as well as pathologies in all organ systems, including cardiovascular disease [9]. Studies have shown that Alzheimer’s disease (AD) patients have fewer intact mitochondria and impaired mitochondrial function attributed to the loss or dysfunction of specific ETC enzymes, and that dysfunctional ETCs trigger ROS accumulation and exacerbate oxidative stress [10]. In addition, excess ROS causes cellular damage and accelerates skin aging [11]. Therefore, the dynamic balance of ROS is a core aspect of maintaining cellular homeostasis, as well as a key driver of a variety of aging-related diseases. Thus, targeting the ROS regulatory network may provide a new strategy for intervening in related pathological processes.

#### mtDNA mutations

The mitochondrial genome is a small circular molecule of approximately 16,500 bp, with multiple copies in each cell (copy numbers range from 1000 to 10,000), and its encoded products are directly involved in the composition of the ETC complex. Studies have shown that mtDNA can lead to ETC dysfunction by accumulating oxidative damage, which, in turn, induces the aberrant generation of ROS, forming a positive feedback loop that ultimately accelerates the aging process. mtDNA damage is related to the p53 signaling pathway, telomere shortening, and other aging-related mechanisms, further exacerbating the cellular senescence phenotype [12]. Decreased mitochondrial autophagic activity in senescent individuals may be an important mechanism by which mtDNA mutations show age-dependent accumulation [13]. Further studies have shown that mitochondrial dysfunction and mtDNA damage are prevalent in the retinal pigment epithelium of patients with age-related macular degeneration (AMD), and this damage leads to oxidative stress, the collapse of energy metabolism, and inflammatory responses [14]. Additionally, mitochondrial damage leads to mtDNA leakage, the activation of the cGAS-NLRP3 inflammatory pathway and the complement system, the exacerbation of vitreous membrane wart deposition, and chronic inflammation, ultimately leading to the characteristic pathological changes in AMD [15]. mtDNA is associated with energy metabolism, and damage usually leads to an inadequate energy supply. For example, during aging, mtDNA defects cause a decrease in subcutaneous white adipose tissue volume, decreasing lipid storage capacity and triggering systemic lipid metabolic stress [16]. Meanwhile, mtDNA defects lead to excessive ROS and ATP depletion, shifting vascular smooth muscle cells from apoptosis to necrosis and accelerating atherosclerosis [17].

#### Abnormal energy metabolism

Adenosine monophosphate-activated protein kinase (AMPK) is a core regulator of cellular energy metabolism and plays multiple roles in the aging process. AMPK, as a central integrator of aging-related signals, is capable of regulating mitochondrial homeostasis, delaying aging-related damage, reprogramming energy metabolism, and exerting aging effects. AMPK is activated in all eukaryotic organisms with impaired mitochondrial function or ATP synthesis [18], which enhances oxidative metabolism, inhibits inefficient energy expenditure, reduces glycolysis-dependence in M1 macrophages, and attenuates mitochondrial oxidative damage. It also optimizes energy partitioning through central and peripheral tissue linkages [19].

AMPK activation decreases with aging, which exacerbates mitochondrial dysfunction and energy metabolism imbalances, leading to organismal aging [20]. When AMPK activity decreases, mTOR activity increases. mTOR is a downstream target molecule of AMPK phosphorylation, and the two maintain metabolic homeostasis antagonistically, forming a dynamic yin and yang regulatory network [21]. AMPK was reported to be activated under hypoxic or energetic stress conditions and regulate the secretory phenotype associated with aging by directly inhibiting the mTOR pathway [22]. The sustained activation of AMPK leads to increased ROS levels, dysregulated mitochondrial quality control, aberrant decreases in mtDNA copy numbers, and the excessive inhibition of mTOR, which impairs mitochondrial dynamic homeostasis and reduces mitochondrial stress adaptation [23]. Therefore, the relationship between AMPK and mitochondrial function shows a typical inverted U-shaped curve, moderate activation is beneficial, but weakened or sustained regulation may be harmful. AMPK not only occupies a key position in energy metabolism, but it is also a potential target for anti-aging interventions. Thus, AMPK should be regulated to circumvent its potential negative effects on mitochondrial function by precisely controlling tissue-specific differences, the degree and timing of activation, and dynamic monitoring of the cellular energy status.

#### Reduced biosynthesis

Mitochondrial biosynthesis refers to the process by which cells maintain their functions through mtDNA replication, respiratory chain protein synthesis, and mitochondrial proliferation. The core regulators include Peroxisome proliferator-activated receptor γ coactivator 1α (PGC-1α), Nuclear Respiratory Factor 1 (NRF1), and mitochondrial transcription factor A (TFAM). Among them, peroxisomal proliferation is the most important. PGC-1α is the master regulator of mitochondrial biosynthesis. It can activate NRF1 and TFAM, mtDNA transcription, and the gene expression of respiratory chain proteins, directly promoting mitochondrial generation and oxidative metabolism. However, PGC-1α expression was found to be downregulated during aging, directly leading to the overall repression of mitochondrial function-related genes and significantly disrupting neuronal energy homeostasis [24]. In addition, in the skeletal muscle of aged mice, PGC-1α overexpression was shown to reverse age-associated decline in mitochondrial translational capacity, restore ETC activity, and induce the transcription of mitochondrial unfolded protein response genes, which help to remove misfolded proteins and maintain mitochondrial protein homeostasis. In contrast, PGC-1α knockdown exacerbated dysfunction, leading to reduced biogenesis, decreased respiratory efficiency, ROS accumulation, and potentially accelerated aging and neurodegenerative pathologies [25].

## Mitochondrial autophagy and its mechanisms

### Autophagy process and its biological significance

**Fig. 2:**
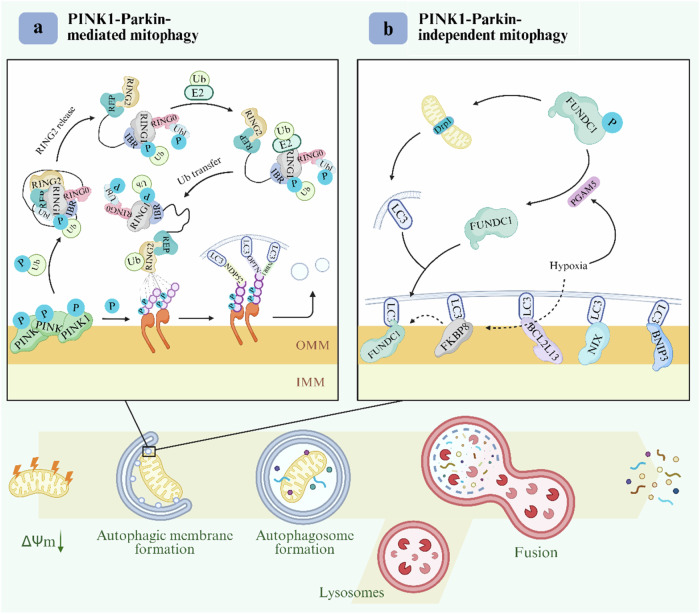
Mitochondrial autophagy process and mechanism. Mitochondrial autophagy proceeds through four stages: membrane potential dissipation of mitochondria-mitochondrial autophagosome formation-autophagosome fusion with lysosome-mitochondrial contents are degraded by lysosomes. **a** PINK1-Parkin‐mediated mitophagy. When mitochondria are damaged, the input of PINK1 is hindered and accumulates on the outer mitochondrial membrane. PINK1 then phosphorylates Ub, which then binds to RING1, resulting in the release of RING2 and the exposure of the E2 interaction surface in RING1. RING2 then receives ubiquitin from E2 and transfers it to the substrate. Parkin is activated to ubiquitinate mitochondrial substrates on OMM, allowing LC3 attached to autophagosomes to be recruited through autophagy adaptors OPTN and NDP52, among others. **b** PINK-Parkin-independent mitophagy. FUNDC1, NIX, BNIP3, BCL2L13, and FKBP8 can bind LC3 alone to mediate mitochondrial autophagy. During reticulocyte maturation, the autophagic receptors NIX, FUNDC1, and BNIP3 are strongly activated, resulting in increased receptor levels on the OMM. Among them, BNIP3 can induce mitochondrial rupture and promote the separation of damaged mitochondria by promoting Drp1. At the same time, BNIP3 recruits Parkin to mitochondria and activates mitochondrial autophagy. Furthermore, hypoxia enhances PGAM5-mediated FUNDC1 dephosphorylation, while FUNDC1 dephosphorylation and mitochondrial fission mediated by FUNDC1-Drp1 complex binding jointly promote mitochondrial autophagy.

Mitochondrial autophagy (mitophagy) is a sophisticated quality control process in which cells selectively remove damaged mitochondria through autophagy. Its core function is to maintain the stability of the mitochondrial network and balance in the intracellular environment. The process follows a strict staged program, involving the dissipation of the membrane potential of mitochondria, the formation of mitochondrial autophagosomes, the fusion of autophagosomes with lysosomes, and the degradation of mitochondrial contents by lysosomes [26]. In addition, mitochondrial autophagy can specifically remove mitochondria with respiratory chain defects due to mtDNA mutations or ROS overload to achieve quality control, as well as the corresponding energy requirements or metabolic re-editing, and the removal of damaged mitochondria to regulate their numbers [27]. Moreover, mitochondrial dysfunction is a driver of aging, and decreases in mitochondrial autophagy directly drive aging pathology. For example, during aging, the decline in mitochondrial autophagy leads to the accumulation of damaged mitochondria, and these dysfunctional mitochondria produce excessive ROS, which not only causes oxidative damage but also acts as signaling molecules to participate in the regulation of aging, resulting in a cycle of mitochondrial damage-ROS production-more damage [28]. Therefore, mitochondrial autophagy is a central hub connecting organelle health and organism aging, and the precise analysis of its molecular mechanism will provide a revolutionary perspective for anti-aging therapy (Fig. 2).

### Autophagy mechanisms

Mitochondrial autophagy occurs through many different but interrelated mechanisms. These can be categorized into ubiquitin-dependent and ubiquitin-independent pathways.

#### Ubiquitin-dependent pathway

In the ubiquitin-dependent pathway, damaged mitochondria are recognized and cleared through the PINK1/Parkin signaling pathway, whose core components include PTEN-inducible kinase 1 (PINK1), Parkin, and the ubiquitin chain [29]. PINK1 is the initiating sensor and main regulator of the pathway, and utilizes kinase activity to convert mitochondrial damage signals into autophagy instructions [30]. In the normal state, PINK1 is imported into the inner mitochondrial membrane via the TOM/TIM complex. Upon the loss of mitochondrial membrane potential, PINK1 accumulates in the outer membrane and is activated to phosphorylate ubiquitin to recruit Parkin [31]. Loss-of-function mutations can lead to defective mitochondrial autophagy and early-onset Parkinson’s disease (PD) [32, 33]. Parkin, as a downstream effector of PINK1, consists of an N-terminal ubiquitin-like structural domain (UBL), a Parkin repressor element (REP), and a C-terminal really interesting new gene 2 (RING2) structural domain or C-terminal really interesting new gene 2 (RING2) structural domains [34]. Its activation relies on a PINK1-mediated phosphorylation cascade. Phosphorylation releases UBL and RING1 structural domains from autoinhibition, inhibits the catalytic activity of RING2, and ultimately leads to the construction of the ubiquitin chain by Parkin on the outer mitochondrial membrane protein [35, 36]. The ubiquitin chain marks damaged mitochondria as “to-be-degraded” targets in a specific modification pattern, and they are recognized and cleared by autophagy, which is essential for maintaining mitochondrial homeostasis [37]. Autophagy receptors (p62, NBR1, OPTN, and NDP52) ensure smooth autophagy by binding both cargo and ubiquitin chains [38]. Notably, NDP52 and OPTN can be directly recruited by PINK1 to achieve Parkin-independent mitochondrial autophagy [39]. Activation of the PINK1/Parkin pathway involves three key components: mitochondrial mass monitoring, ubiquitin signaling, and autophagosome recruitment [40]. In summary, PINK1 detects mitochondrial dysfunction and generates ubiquitin phosphate as an autophagy signal. Parkin amplifies and ubiquitinates outer mitochondrial membrane proteins, and ultimately directs the removal of damaged mitochondria by autophagy [41, 42].

#### Ubiquitin-independent pathway

The ubiquitin-independent pathway regulates mitochondrial autophagy through three mechanisms: (1) direct receptor recruitment to autophagosomes: outer mitochondrial membrane receptors, such as BNIP3, NIX, BCL2L13, and FKBP8, directly bind to the autophagy marker protein, LC3, through their LIR motifs (LC3-interacting region), bypassing the PINK1/Parkin ubiquitylation pathway, and mediate the clearance of damaged mitochondria; (2) stress-induced receptor activation: during hypoxia or mitochondrial damage, receptors (FUNDC1) undergo dephosphorylation or conformational changes to mitigate the inhibitory state to enhance LC3 binding capacity and initiate autophagy; and (3) receptor synergistic network regulation: some receptors (FKBP8 and FUNDC1) can form an interoperative network to finely regulate mitochondrial quality control [43]. Among them, in the BNIP3/NIX pathway, BNIP3 and NIX, which are homologous proteins of BCL2, directly recruit LC3 through LIR motifs without the involvement of ubiquitination. This pathway efficiently clears mitochondria in programmed autophagy (erythrocyte maturation) and in response to acute stress [44, 45]. In contrast, the FUNDC1 pathway anchors autophagosomes to mitochondria by binding LC3 via LIR, and its activity is dynamically regulated by phosphorylation, phosphorylation inhibits LC3 binding in the basal state, and dephosphorylation activates autophagy during hypoxia [46, 47].

### Metabolic regulation of autophagy

In addition to the PINK1/Parkin signaling pathway and receptor-dependent pathways described above, metabolites such as iron, calcium, and NAD⁺ regulate mitochondrial autophagy by targeting key molecular nodes. Iron is the most prevalent metal in the mitochondrial matrix, contributing to the complex redox chemistry that facilitates the electron transport chain. Iron levels are closely linked to mitochondrial energy metabolism. Iron overload inhibits mitochondrial autophagy, and excess iron exacerbates ROS bursts, inhibits PINK1 stability and Parkin translocation, and leads to the accumulation of damaged mitochondria. In contrast, iron deficiency activates mitochondrial autophagy, and a low-iron environment induces FUNDC1 dephosphorylation, enhances the ability to bind the LIR motif to LC3, and promotes mitochondrial autophagy. In addition, iron chelators (Deferiprone) promote FIS1 localization in mitochondria through SENP3-mediated FIS1 de-SUMOization, driving mitochondrial division and autophagy [48, 49]. Calcium ions (Ca^2+^) are one of the most versatile signaling molecules and important regulators of mitochondria. RHOT1/RHOT2 (Miro proteins) act as Ca^2+^ sensors, coordinating mitochondrial motility and the PINK1/Parkin pathway. At normal Ca^2+^ concentrations, Miro proteins anchor mitochondria to kinesins and maintain organelle distribution. In cases of excess mitochondrial Ca^2+^ uptake, mitochondrial membrane potential collapses and inhibits mitochondrial autophagy, leading to cell death [50]. Nicotinamide adenine dinucleotide (NAD⁺) is a key coenzyme widely present in living cells. Its biological function is mainly reflected in two aspects. On the one hand, it acts as a coenzyme of oxidoreductase and participates in the electron transfer process, and on the other hand, it acts as a co-substrate for a variety of enzymes. Studies have shown that the cellular concentration of NAD⁺ changes significantly during the aging process, whereas the mitochondrial autophagy process can be affected by regulating the NAD⁺ metabolic pathway, which, in turn, has a modulating effect on lifespan [51]. NAD⁺ and mitochondrial autophagy are bidirectionally regulated. NAD⁺ promotes autophagosome formation and removes damaged mitochondria by activating nutrient-sensing pathways (AMPK/mTOR) and enhancing autophagic core proteins (ULK1 and the PI3K-III complex). Meanwhile, normal autophagy can maintain NAD⁺ homeostasis, whereas defective autophagy leads to mitochondrial dysfunction, triggering excessive ROS and DNA damage, and the overactivation of PARP/SIRT accelerates NAD⁺ depletion, ultimately leading to cell death. Further studies showed that supplementation with NAD⁺ precursors (NR/NMN) rescued the survival of autophagy-deficient cells and prolonged the healthy lifespan of model organisms [52]. The above metabolites dynamically regulate mitochondrial autophagy efficiency through energy-sensing organelle interactions. Although the specific mechanism of action has not been fully elucidated, these findings reveal the potential association between mitochondrial autophagy and cellular metabolism and provide a new research direction for treating mitochondrial autophagy dysfunction.

## The association between mitochondrial autophagy and anti-aging

### Double-edged sword effect of autophagy in aging

**Fig. 3:**
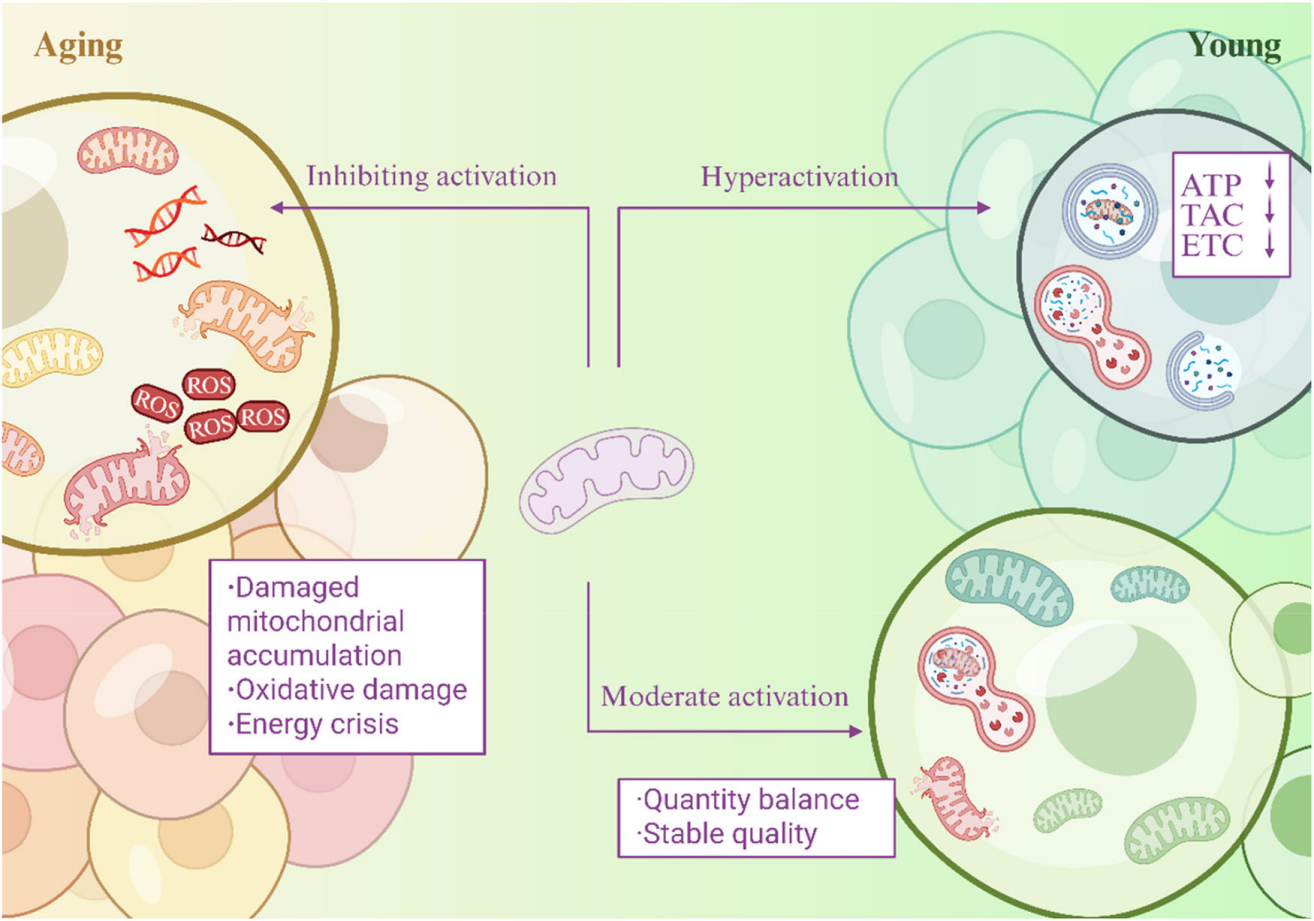
Dual roles of autophagy: protective barrier and pathology driver.

Mitochondrial autophagy maintains cellular homeostasis by selectively removing damaged mitochondria; however, it exhibits a loss of dynamic equilibrium during the aging process. The moderate activation of mitochondrial autophagy can precisely regulate the balance between mitochondrial quality and quantity to delay aging [53]. For example, ubiquitin-dependent autophagy mediated by the PINK1/Parkin pathway recognizes mitochondria with dissipated membrane potential and recruits LC3 to form an autophagosome via the OPTN/NDP52 receptor to remove defective respiratory chain units, while receptors such as BNIP3/FUNDC1 directly anchor LC3 via LIR motifs in response to cellular stress signals to maintain the balance of mitochondrial numbers [28]. In contrast, mitochondrial autophagy efficiency may decrease during aging, leading to the accumulation of damaged mitochondria, which exacerbates cellular damage, creating a vicious cycle. For example, defective mitochondrial autophagy in AD patients leads to the accumulation of damaged mitochondria, and the inability to efficiently scavenge dysfunctional mitochondria exacerbates oxidative damage and an energy crisis [54]. In addition, the over-activation of mitochondrial autophagy in some cases may lead to excessive mitochondrial clearance, affecting energy availability. For example, in *Drosophila melanogaster* mtDNA mutants, PINK1 overexpression triggered excessive autophagy that removed greater than 90% of the mitochondria, leading to a collapse of ATP synthesis with stagnant larval development [55]. In summary, mitophagy acts as both a shield (removing damage to maintain homeostasis) and a spear (accelerating pathology when dysregulated) in aging. Future studies need to focus on tissue-specific thresholds and dynamic equilibrium nodes to achieve precise interventions (Fig. 3).

### Mechanisms of autophagy regulation during aging

#### Gene regulation

**Fig. 4:**
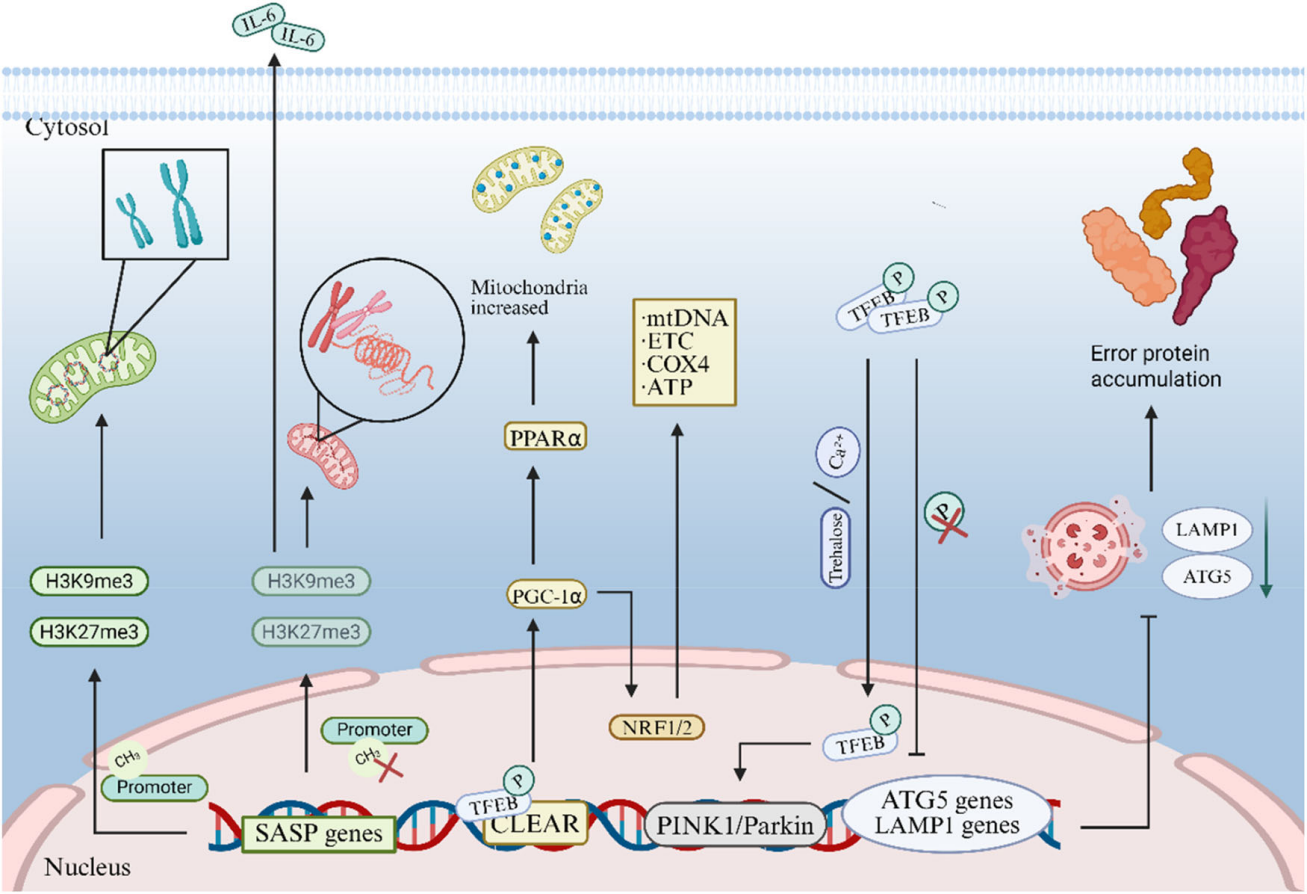
Gene regulation.

Mitochondrial gene regulation is critical for maintaining respiratory chain function and redox homeostasis, and its disruption accelerates aging and degenerative disease processes by disrupting energy metabolism and increasing oxidative damage [56, 57]. Gene regulation is multidimensionally dysregulated during aging (Fig. 4). At the epigenetic level, the episodic silencing of key mitochondrial genes (elevated DNA methylation) exacerbates energy metabolism disorders, ROS accumulation, and cellular senescence, further accelerating epigenetic drift [58], and disturbed methylation patterns can lead to transposon activation and DNA damage accumulation, exacerbating cellular functional decline [59]. Meanwhile, aberrant histone modifications (H3K27me3 deletion) directly lead to the aberrant activation of senescence-associated secretory phenotypic (SASP) genes pro-inflammatory factors such as interleukin (IL-6), driving chronic inflammation [60]. At the transcriptional level, compared with normal cells, the key autophagy-lysosomal master transcription factor (TFEB) retains the cytoplasm and is phosphorylated and inactivated more than 3-fold longer in senescent cells, leading to the expression of autophagy genes (ATG5 and LAMP1) is downregulated by 50%, causing a decrease in the expression of lysosomal and autophagy-related genes. Thus, erroneous proteins and damaged organelles cannot be effectively cleared, causing a cycle of metabolic disorders and dysregulated gene expression, which accelerate the occurrence of aging and related diseases [61]. Mitochondrial autophagy releases Ca²⁺, which induces TFEB activation and promotes nuclear translocation. TFEB entry into the nucleus directly upregulates PINK1 and Parkin expression and promotes mitochondrial biogenesis via the PPARα/PGC-1α axis [62]. Martini-Stoica et al. demonstrated that the activation of TFEB by drugs (alginin) promotes its nuclear translocation, restores lysosomal function, significantly reduces pathological inclusions of α-synuclein, and ameliorates behavioral deficits in animal models [63]; however, the over-activation of TFEB may lead to lysosomal overconsumption or autophagic cell death [64]. Researchers have found that DNA methylation changes are involved in senescence regulation, affecting cellular function through the methylation disruption of developmentally critical genes (imprinted genes), as well as inducing genomic instability (transposon activation) associated with senescence [65]. In contrast, the naked mole rat, which has specific epigenetic regulation, maintains an “ageless” phenotype through multiple mechanisms, including the LHX3-POU transcriptional network, the precise regulation of methylation of developmental genes, and transposon silencing [66]. Although methylation changes are widely observed during aging (small but widespread CpG/CpH site alterations), direct evidence that these changes are a driver rather than a concomitant phenomenon of aging is lacking [67].

#### Mitochondrial quality control

**Fig. 5:**
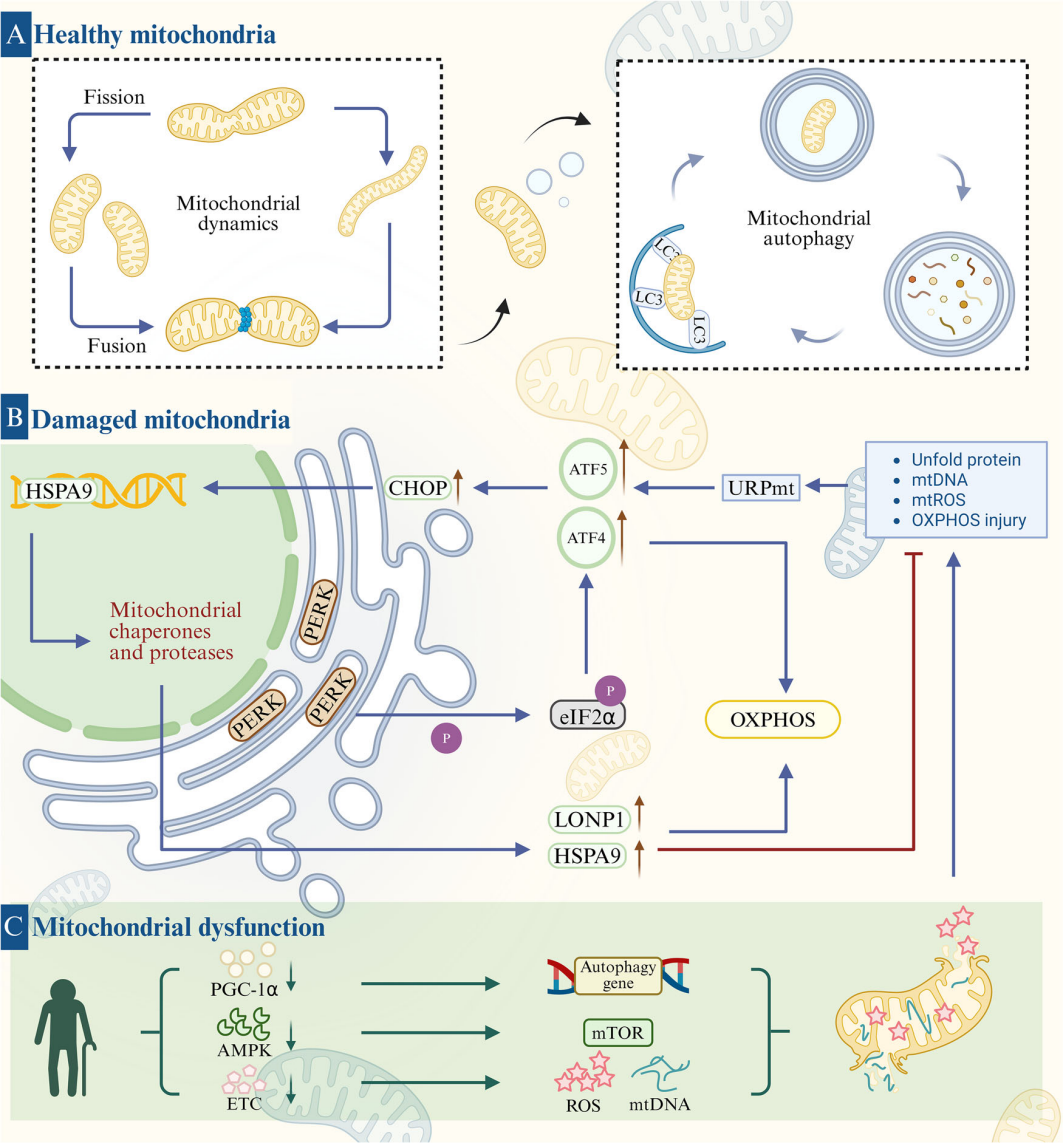
Mitochondrial quality control. **a** Healthy mitochondria. The damaged parts of mitochondria split from the healthy parts by split proteins and are encapsulated by autophagosomes for mitochondrial autophagy. While the healthy part of the mitochondria fuses with other mitochondria to achieve its anti-aging function. **b** Damaged mitochondria. Protein misfolding, mtROS and mtDNA leakage, and oxidative phosphorylation damage can all activate URPmt. URPmt initiation induces ATF4 to bind to CHOP, while ATF5 directly acts on the mitochondrial chaperone promoter, upregulating the expression of HSPA9 and LONP1 to rebuild homeostasis in mitochondria. In addition, PERK further activates the expression of ATF4 by phosphorylating eIF2α, thereby repairing oxidative phosphorylation damage. **c** Mitochondrial dysfunction. Aging causes decreased expression of PGC-1α and AMPK and impaired ETC, which in turn affects the normal transcription of autophagy genes, upregulates mTOR expression, and leaks ROS and mtDNA, ultimately exacerbating mitochondrial dysfunction.

Mitochondria are important organelles within eukaryotic cells. Mitochondrial quality control (MQC) has evolved in response to a variety of physiological signals and external stimuli to maintain mitochondrial health and function through a multilayered synergistic mechanism. These include mitochondrial proteostasis maintenance, the regulation of mitochondrial dynamic homeostasis, mitochondria-derived vesicle formation, and mitochondrial autophagy (Fig. 5) [68]. Senescence leads to synergistic MQC failure and triggers cascading damage. The reduced expression or activity of PINK1 and Parkin in senescent cells restricts the clearance of damaged mitochondria, which triggers a variety of diseases. For example, in PD patients, PINK1/Parkin mutations cause impaired mitochondrial autophagy and mitochondrial accumulation in dopamine neurons, leading to oxidative stress and cell death [35]. The mitochondrial unfolded protein response (UPRmt) is a key mechanism for maintaining mitochondrial proteostasis, and mitochondria in aging organisms experience elevated proteotoxic stress (misfolded protein accumulation), UPRmt depletion, and the downregulation of molecular chaperones (HSPA9/HSPD1) and proteases (LONP1), which results in the accumulation of misfolded proteins in mitochondria that inhibit autophagy [69]. MQC can be positively regulated by mitochondrial autophagy, and autophagy cooperates with UPRmt to build a repair-clearance bipartite axis, UPRmt mainly repairs mitochondria damaged in the early stage, and if it fails to do so, it promotes the autophagy of PINK1/Parkin to clear irreparable mitochondria, which can help to restore OXPHOS function and maintain mitochondrial homeostasis. Parkin expression (or the lack of expression) does not interfere with the levels of activated UPRmt. Parkin expression and UPRmt activation are independently initiated but have complementary functions. Together, they constitute a double-insurance mechanism for mitochondrial quality control [70]. Wenshu Cong et al. reported that cobalt oxide nanoparticles modified with dimercaptosuccinic acid could serve as novel UPRmt activators. The metabolic regulation and protein homeostatic network enhancement by activating UPRmt significantly prolonged the healthy lifespan in the cryptic rod nematode (*C. elegans*) model [71]. The aging process is often accompanied by an imbalance in mitochondrial dynamics, which causes mitochondrial dysfunction, leading to cell fate abnormalities and a range of aging-related diseases [72]. Mitochondrial autophagy can couple with kinetic balances to optimize quality control. For example, autophagy targets the removal of excessive DRP1-mediated division products, and moderate fusion prevents autophagic overactivation [73]. Further studies revealed that mitochondrial GTP metabolism controls reproductive senescence in *Cryptomeria hollisteri* by promoting mitochondrial fission, preventing the aggregation of damaged mitochondria, and enhancing the efficiency of mitochondrial autophagy, which subsequently maintains mitochondrial mass in oocytes [74]. Thus, mitochondrial autophagy reverses senescence-associated MQC failure through the triple action of removing irreparable mitochondria, synergizing UPRmt repair, and regulating kinetic homeostasis.

#### Protein regulation

**Fig. 6:**
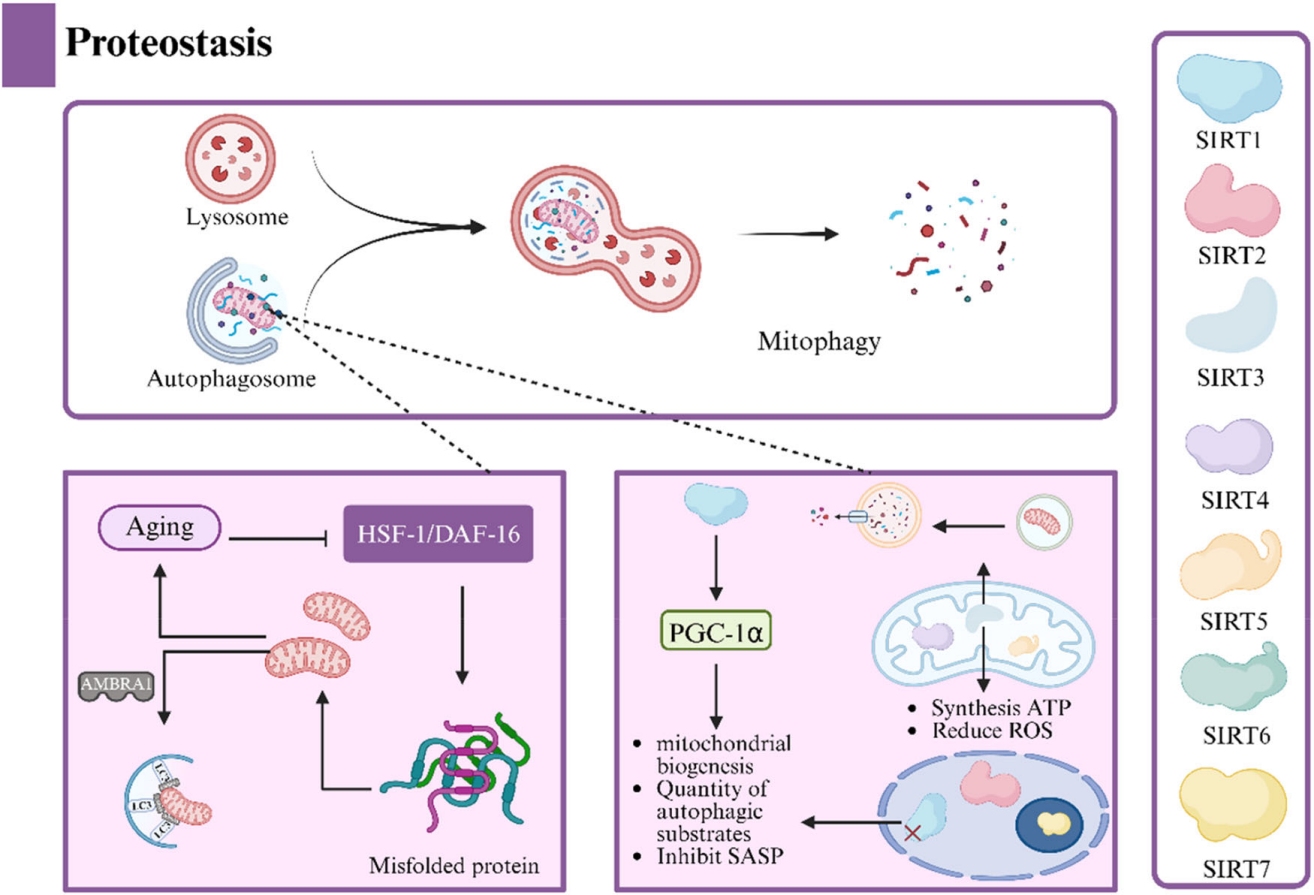
Protein homeostasis network.

Proteostasis (protein homeostasis) maintains cellular function and delays aging by safeguarding proper protein folding and degradation. It is a key barrier to maintaining cellular function, and its decline with age is a central driver of aging (Fig. 6) [75]. The recession of long-life signaling pathways, such as Heat Shock Factor 1/Dauer Formation 16, during aging leads to the accumulation of misfolded proteins and increases protein damage, which accelerates aging [76]. In addition, the increased oxidative modification of proteins associated with aging and decreased clearance capacity synergize to cause protein aggregation problems, leading to the ineffective clearance of misfolded proteins and the development of functionally acquired toxicity disorders such as PD, Huntington’s disease, and AD [77]. Researchers found that mitochondrial autophagy improves protein homeostasis via a dual pathway. On the one hand, autophagy directly removes the sources of damage through protein mediation. For example, autophagy and beclin 1 regulator 1 (AMBRA1), a central hub for mitochondrial quality control and protein homeostasis, removes toxic protein-containing mitochondria by recruiting LC3 through PINK1/Parkin-dependent and non-dependent pathways. However, aging causes its dysfunction, leading to neuronal death and neurodegeneration [78]. On the other hand, autophagy can synergize with the sirtuin family (Table 1) to delay aging by regulating protein acetylation homeostasis, energy metabolism, and oxidative stress [79]. SIRT1, located in the nucleus and cytoplasm, regulates mitochondrial biosynthesis, and indirectly affects the number of autophagy substrates through deacetylating transcription factors (PGC-1α) [80]. Its downregulation enhances immune surveillance (inhibits the secretion of senescence associated secretory phenotype factors) during acute aging [150]. SIRT3, located in mitochondria, directly manages beta oxidation, ATP synthesis, and ROS scavenging [152]. Further studies revealed that SIRT3 overexpression significantly activated autophagy, reversed decreases in mitochondrial membrane potential induced by advanced glycosylation end products (AGEs), and maintained mitochondrial integrity [153]. Mitochondrial autophagy blocks the cycle of protein homeostasis through AMBRA1-mediated clearance and the synergistic repair of sirtuins, providing a new concept for improving mitochondrial quality as well as slowing the aging process.

**Table 1:** Sirtuin protein family.

Protein name	Subcellular localization	Core function	Disease association	References
SIRT1	Nucleus, cytoplasm	Deacetylates transcription factors (forkhead box protein, peroxisome proliferator-activated receptor γ coactivator 1α, tumor protein 53); regulates autophagy, mitochondrial biosynthesis, and inhibits senescence associated secretory phenotype secretion; Nicotinamide adenine dinucleotide-dependent deacetylases	Neurodegenerative diseases: Alzheimer’s disease (autophagy disorders); metabolic diseases: insulin resistance, obesity; aging: stem cell depletion	[79, 80, 150]
SIRT2	Cytoplasm, nucleus	Deacetylated microtubule proteins; maintains neuronal homeostasis against oxidative stress and inflammatory damage; regulates cellular autophagy, energy metabolism, and aging-related phenotypes	Parkinson’s disease (PD): alpha-synuclein aggregation; cancer: enhanced breast cancer metastasis; chronic inflammation: arthritis	[151]
SIRT3	Mitochondrial matrix	Deacetylates electron transport chain complexes (complex I/II); Activates superoxide dismutase 2 antioxidant enzyme; regulates fatty acid beta oxidation, ketogenesis	Aging-related diseases: mitochondrial dysfunction; metabolic syndrome: diabetic cardiomyopathy; cancer: tumor metabolic reprogramming	[152, 153]
SIRT4	Mitochondrial matrix	Inhibits adenosine diphosphate ribosyltransferase activity; inhibits pyruvate dehydrogenase inhibits glycolysis; regulates insulin secretion	Diabetes: defective insulin secretion; obesity: lipid accumulation	[154]
SIRT5	Mitochondrial matrix, cytoplasm	Modifies desuccinylation/malonylation; regulates the urea cycle, Tricarboxylic acid cycle enzymatic activity	Non-Alcoholic Fatty Liver Disease: lipid accumulation; Alzheimer’s disease: oxidative stress injury	[155]
SIRT6	Nucleus	Deacetylates histone Histone H3 lysine 9/Histone H3 Acetyl; maintains telomere stability, DNA damage repair; inhibits nuclear sactor-kappa B reduces inflammation	Genomic instability, accelerated aging phenotype; cardiovascular disease: atherosclerosis; diabetes: dysregulation of gluconeogenesis	[108]
SIRT7	Nucleolus	Regulates rDNA transcription; activates RNA polymerase I	Cardiomyopathy: direct inhibition of GATA-binding protein 4; aging: nucleolar stress	[156]

#### Activation of the inflammatory response

**Fig. 7:**
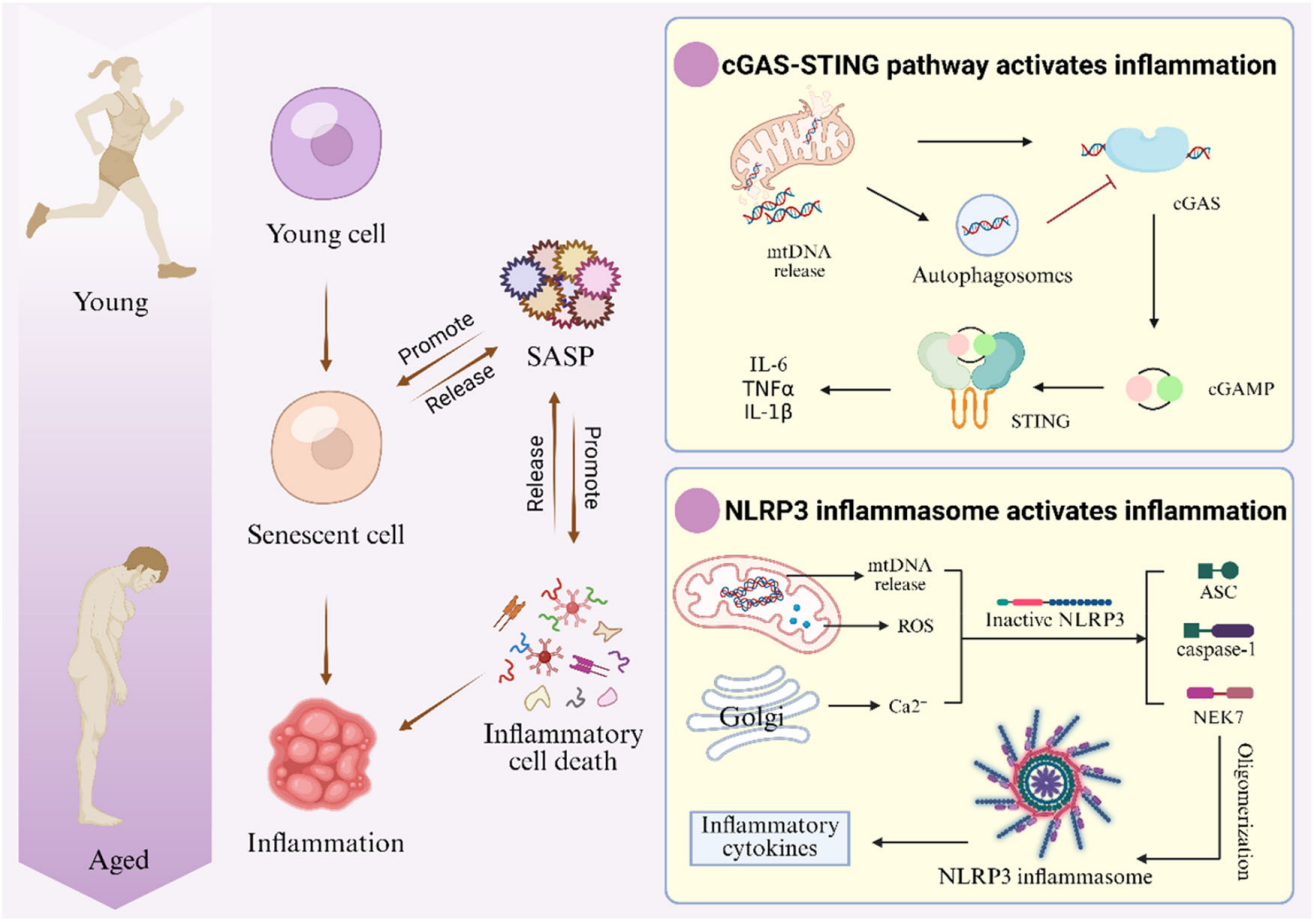
Links between aging and inflammation.

During aging, almost all cells in the body undergo senescence, and senescent cells drive chronic inflammation through a SASP: they secrete factors such as IL-6, tumor necrosis factor (TNF)-α, and transforming growth factor (TGF)-β, which recruit immune cells to remove senescent cells. However, when immune escape occurs, the continued release of SASP triggers “inflammaging”, leading to tissue fibrosis, organ damage, and age-related diseases (Alzheimer’s disease and atherosclerosis), while inflammatory signaling feedback promotes more cellular senescence, creating a self-reinforcing cycle [81, 82]. Mitochondria are central to this triangular cycle: dysfunctional mitochondria release molecules such as mtDNA and cardiolipin, which activate SASP and systemic inflammation via cGAS-STING and the NLRP inflammasome, a dual pathway that further damages mitochondria (Fig. 7) [2, 83, 84]. SASP is also associated with most of the non-autonomous effects observed in senescent cells, including inflammation, senescence enhancement, and paracrine senescence [85]. NLRP3, which is a mitochondrial damage sensor, is the most strongly associated with senescence in the many inflammatory vesicles (Table 2 Introduction to inflammatory vesicles) [86]. In contrast, defective mitochondrial autophagy induces ROS overproduction [87], mtDNA leakage, and the exposure of cardiolipin, which are signals that activate NLRP3, leading to the onset of inflammatory responses [88–90]. Thus, intact and functioning mitochondria are the primary gatekeepers controlling genetic, metabolic, and inflammatory homeostasis [3]. Mitochondrial autophagy removes damaged mitochondria, blocks mtDNA leakage, degrades oxidized phospholipids and removes inflammatory triggers [91]. The specific knockdown of mitochondrial transcription factor A (TFAM) decreased mtDNA synthesis in mouse macrophages and significantly inhibited NLRP3 activation, thus demonstrating that mtDNA synthesis is the rate-limiting step in NLRP3 activation [92]. In addition, lowering mtROS levels also inhibited TFAM-mediated mtDNA synthesis and NLRP3 activation [93]. Mitochondrial autophagy not only secures the ATP supply and prevents ATP/ADP imbalances to weaken NLRP3 autoinhibition [94], but also wraps cytoplasmic mtDNA to form mtDNA-trapping vesicles [95], which prevents it from contacting cGAS to block the cGAS-STING pathway, inhibiting the release of SASP factor [83, 84]. In conclusion, enhancing mitochondrial autophagy protects the normal function of mitochondria, which helps to ensure their integrity to avoid inflammation caused by the leakage of pro-inflammatory factors, and thus slows aging (Table 3).

**Table 2:** Introduction to inflammatory vesicles.

Protein name	Activation mechanism	Core function	Associated diseases	References
NLRP3	Signals mitochondrial damage: mtDNA leakage, cardiolipin exposure, and reactive oxygen species (ROS) overload. Other signals: ATP, crystals (uric acid/β amyloid), pathogens	Assembly of ASC-caspase-1 complex, activation of interleukin-1β (IL-1β) \ interleukin-18 (IL-18) induction of cellular pyroptosis	Aging-related: Alzheimer’s disease (Aβ activation), atherosclerosis (cholesterol crystallization activation) and type 2 diabetes (pancreatic β-cell damage). Others: gout, CAPS (autoinflammatory syndrome)	[86, 88, 92, 94]
NLRC4	Viral RNA response	Significantly inhibit the activation of NLRP3 and absent in melanoma 2 inflammatory vesicles and reduces the release of IL-1β and IL-18; promotes autophagy through the beclin-1-dependent pathway	Viral infections: negative regulation of type I interferon response by NLRP4 may result in viruses evading immune clearance; autoimmune diseases: exacerbates joint inflammation	[157]
NLRP1	Deregulates recombinant dipeptidyl peptidase 8/9 inhibition; Activates protease cleavage; directly senses dsRNA	Activates caspase-1 to cleave IL-1β precursor	Activation by *Bacillus anthracis* triggers immune defenses; autoimmune disease: hyperactivation of NLRP1 may be associated with chronic inflammation	[158]
AIM2	Directly binds dsDNA	Modulates autoimmune tolerance; maintains genomic stability; anti-infection immune defense	Autoimmunity: systemic lupus erythematosus; infection: HIV infection; aging-associated: cytoplasmic mtDNA accumulation drives inflammation	[159]
Pyrin	Rho GTPase inactivation in response to bacterial toxins	Immune defense, inflammatory regulation, autoimmune homeostasis	Autoinflammatory diseases: familial Mediterranean fever; pathogenic infections: salmonella infection	[160]

**Table 3:** Mitochondrial autophagy and senescence regulatory targets.

Mechanism classification	Name of target or pathway	Regulatory mechanism	Related phenotypes	References
Gene Regulation	Epigenetic silencing	Elevates DNA methylation of mitochondrial genes; aberrantly activates the senescence-associated secretory phenotypic (SASP) gene due to histone H3 lysine 27 trimethylation deletion	Decline in lysosomal function; pathologic inclusion body accumulation of alpha-synuclein; metabolic disorders	[61–63]
	Transcriptional level	TFEB retention in the cytoplasm and 50% downregulation of autophagy genes during aging; upregulation of PINK/Parkin in the nucleus upon activation promotes mitochondrial biogenesis	Impairs energy metabolism; chronic inflammation	[58–60]
MQC	PINK/Parkin pathway	Reduces activity in aging and impairs the clearance of damaged mitochondria; synergizes with mitochondrial unfolded protein response (UPRmt) to form a “repair-clearance” bi-axis	Mitochondrial accumulation in PD dopamine neurons; oxidative stress cell death	[68, 69]
	UPRmt	Chaperones and proteases are downregulated during senescence; Co3O4NPs activate UPRmt neurons to extend lifespan	Misfolded protein accumulation inhibits autophagy; extends healthy nematode lifespan	[70, 72]
	Kinetic homeostasis	Dynamin-related protein 1 mediates removal of excessive fission products by autophagy; mitochondrial guanosine triphosphate metabolism promotes fission-enhanced autophagy efficiency	Decreases mitochondrial mass in oocytes; accelerates nematode reproductive senescence	74, 75]
Protein regulation	AMBRA1 mediates clearance	Wrapping of toxic protein-containing mitochondria via PINK1/Parkin-dependent/independent pathway; dysfunction during senescence	Neuronal death; oligomyopathy	[78, 79]
	Sirtuins synergize to repair	SIRT1: Deacetylated PGC-1α regulates mitochondrial biosynthesis; SIRT3: management of β oxidation/ROS scavenging to reverse AGE damage	Protein aggregation diseases; Metabolic network reconstruction to delay aging	[80, 83, 84]
Inflammatory response activation	mtDNA-cGAS-STING pathway	Autophagy defects lead to mtDNA leakage and activation of the cGAS-STING pathway; autophagosomes form mtDNA trapping vesicles to block the pathway	Sustained release of SASP factors; systemic inflammatory senescence	[91, 92, 104]
	NLRP3 inflammatory vesicles	ROS overload/mtDNA leakage/cardiolipin exposure activates NLRP3; Inhibits mtDNA synthesis by mitochondrial transcription factor A knockdown, blocking activation	Tissue fibrosis and organ damage; atherosclerosis progression	[95, 97, 101]
	Mitochondrial autophagy; anti-inflammatory	Removes damaged mitochondria blocks mtDNA leakage; eliminates inflammatory triggers by degrading oxidized phospholipids	Delays age-related diseases	[100]

## Regulating mitochondrial autophagy to fight aging

Mitochondrial autophagy is a cellular quality control mechanism and may be a driver of pathological processes, depending mainly on factors such as the extent of damage, cell type, and the strength of pathway activation. Early in the process, aging can be slowed by removing damaged mitochondria; however, with age, mitochondrial autophagy may become a pro-aging process due to lysosomal decline or the erroneous degradation of key proteins [57]. Therefore, intervening in autophagy using rational means is extremely important for delaying aging (Table 4).

**Table 4:** Drugs that exert anti-aging effects by modulating mitochondrial autophagy and their mechanisms.

Drug name	Animal model	Dose and cycle	Mechanism of action	Effect	References
Urolithin A	Clinical trial in elderly people	500–1000 mg/day for 4 weeks	Activates PINK1/Parkin pathway; directly recruits light chain 3 (LC3) to promote autophagosome formation	Significant upregulation of 13 mitochondrial genes in skeletal muscle	[97]
	*Caenorhabditis elegans* cryptic rod nematode	50 μM	Directly activates mitochondrial autophagy markers (lipidated form of LC3)	Lifespan extension by 45.4%	[98]
Sulforaphane (SFN)	Aged mice	1–5 mg/kg for 4–12 weeks	Nuclear factor erythroid 2-related factor 2 nuclear translocation activates PINK1/Parkin pathway	Removes damaged mitochondria and reduces senescence phenotype	[99]
Spermidine	*Drosophila melanogaster*	1 mM continuous feeding	Activates PINK1/Parkin pathway	Significantly restores short-term/intermediate memory	[102]
	AD transgenic mice	3 mM short-term (to 120 days of age) or long-term (up to 290 days of age)	Activates the PINK1/Parkin pathway	Removes mitochondrial debris under Aβ toxicity	[103]
Metformin			Activates AMPK to promote unc-51likeautophagyactivatingkinase1 phosphorylation; upregulates PINK1/Parkin	Promotes autophagosome formation and prolongs lifespan	[105]
NMN	Mouse		Upregulates PINK1/Parkin; SIRT3 deacetylates mitochondrial proteins; activates SIRT1	Median lifespan extension of 15–20%; amelioration of metabolic abnormalities; reestablishment of metabolic networks	[107, 108]
	Alzheimer’s disease nematode	0.1–1 mM short-term: 3–7 days; long-term: 10–14 days	Elevates sirtuin levels; induces mitochondrial autophagy	Significantly improves cognitive decline	[109]
	Rodents (liver/fat/mus-cle)		Promotes nicotinamide adenine dinucleotide biosynthesis, activates peroxisome proliferator-activated receptor γ coactivator 1α	Improves obesity and aging-related abnormal metabolism	[110, 111]
NR	Aged mice	Dietary intervention for 6 weeks or months	Activates UPRmt	Removes misfolded proteins	[113]
Rapamycin			Inhibits mammalian target of rapamycin complex 1 (mTORC1)-triggered mitochondrial autophagy	Improves mitochondrial dysfunction due to aging	[114]
Quercetin	Stem cell model of premature aging		Upregulates PINK1/Parkin	Ameliorates value-added impairment; reduces pro-senescence proteins	[117]
	Mice with premature aging	Combined with dasatinib, 20–50 mg/kg for days to weeks	Upregulates PINK1/Parkin	25% increase in median lifespan	[118]
Goldfinch isoflavin	Ovariectomized rats	50 mg/kg orally once daily for 8 weeks	Directly activates mitochondrial autophagy	Reduces ROS/DNA damage; downregulates SASP	[119]
Psoralen	Aged mice	20 mg/kg for 14 days	Inhibits mTORC1 pathway to activate mitochondrial autophagy	Significantly reduces fasting blood glucose, total cholesterol, low-density lipoprotein and triglyceride levels; improves mitochondrial function	[120]
DMC	Yeast/worm/*Drosophila*		Activates GATA transcription factor, enhances autophagic flow	Extends lifespan and reduces Sequestosome-1 accumulation in Drosophila brain	[121, 122]
Ginkgetin	Aged mice	5 mg/kg administered every 2 days for 2 months or 8 weeks		Blocks cGAS-STING inflammatory pathway	[123]

### Pharmacological modulation

#### Chemical drugs

Urolithin A (UA) is a naturally occurring polyphenol produced in the intestine by ellagitannins and ellagic acid [96]. UA promotes mitochondrial autophagy by modulating the PINK1/Parkin pathway, as well as activating other receptor proteins and directly recruiting LC3 to promote autophagosome formation. Clinical studies have shown that all 13 mitochondria-related genes were significantly upregulated in the skeletal muscle of elderly people after 4 weeks of 500–1000 mg UA administration [97]. Further experiments revealed that the lifespan of the *Cryptococcus hidradii* nematode was significantly extended by 45.4% after treatment with 50 μM UA [98]. Sulforaphane (SFN), mainly derived from cruciferous vegetables, is an NRF2 (NFE2L2) activator. SFN was found to activate the PINK1/Parkin pathway by promoting NRF2 nuclear translocation in aging mice, which removes damaged mitochondria and reduces the aging phenotype [99]. Spermidine is an endogenous metabolite that slows or prevents the progression of neurodegenerative diseases with age [100]. Aging leads to a significant decline in brain levels of spermidine, and supplementation with spermidine activates mitochondrial autophagy, reducing oxidative damage and impaired energy metabolism within neurons [101]. For example, short-term memory and intermediate memory were significantly restored in aged *Drosophila* after spermidine supplementation [102]. Spermidine was shown to promote the formation of mitochondrial autophagosomes in the Aβ toxic environment of AD transgenic mice by activating the PINK1/Parkin pathway, which removes mitochondrial debris in AD models [103]. Metformin is a drug approved for treating diabetes, but it has been found to regulate aging by inhibiting inflammatory pathways and modulating oxidative stress, autophagy, and protein synthesis [104]. Previous studies demonstrated that the core mechanism of metformin in prolonging the lifespan is by activating AMPK, which directly phosphorylates ULK1 (autophagy-initiating kinase) and promotes the formation of autophagy precursors. It also upregulates the expression of PINK1, which promotes the recruitment of Parkin to the mitochondria and labels damaged mitochondria for clearance [105]. NAD⁺ is one of the several key markers that directly affect aging. During aging, NAD⁺ levels decrease significantly, leading to impaired mitochondrial autophagy and shortened lifespan, whereas nicotinamide mononucleotide (NMN), a direct precursor of NAD⁺, reversed this trend by upregulating the PINK1/Parkin pathway [106] and facilitating the clearance of damaged mitochondria (median lifespan was 15–20% longer in different strains of mice) [107]. In addition, NMN is closely related to sirtuins, reducing ROS damage and rebuilding the metabolic network of senescent cells by regulating the precise deacetylation of 39 mitochondrial proteins by SIRT3 [108]. NMN also ameliorates senescence-associated neurovascular function and cognitive deficits by upregulating SIRT1 activity. For example, in a nematode model of AD, NMN significantly ameliorated cognitive decline by elevating sirtuin levels and inducing neuronal mitochondrial autophagy [109]. Researchers found that insufficient NAD⁺ biosynthesis in rodent liver, adipose tissue, and skeletal muscle led to metabolic abnormalities associated with obesity and aging, which were ameliorated by NMN supplementation [110]. Increased levels of NAD⁺ may promote mitochondrial biosynthesis through pathways such as PGC-1α and alleviate energy crises in senescent cells [111]. NAD⁺ metabolites may likewise activate energy-sensing pathways and promote the expression of mitochondrial autophagy-related proteins (ULK1 and BNIP3) to retard senescence [112]. In addition, the NAD precursor nicotinamide riboside (NR) maintains mitochondrial homeostasis and alleviates the aging process by activating UPRmt to remove misfolded proteins [113]. Rapamycin was found to mimic developmental signals (meiotic initiation) to trigger programmed mitochondrial autophagy by inhibiting the mTORC1 pathway, which has very positive therapeutic significance for autophagy dysfunction caused by aging [114].

#### Traditional Chinese medicine and its active ingredients

Anti-aging has become a hot topic in Chinese medicine. Researchers have achieved many good anti-aging effects of flavonoids. Quercetin, a natural flavonoid found in a variety of fruits and vegetables, is a particularly promising treatment for various systemic or degenerative diseases due to its anti-aging, antioxidant, anti-inflammatory and anti-tumor properties [115]. Quercitin was shown to promote mitochondrial autophagy and scavenge damaged mitochondria by upregulating the PINK1/Parkin pathway [116]. Further mechanistic studies showed that quercetin greatly ameliorated senescence-associated proliferative disorders, reduced pro-senescence proteins, and exhibited significant anti-aging effects in a mesenchymal stem cell model of patients with Werner syndrome and Hutchinson-Gilford premature aging syndrome [117]. The combination of quercetin and other drugs also has great anti-aging potential. The median lifespan of premature aging model mice treated with the combination of quercetin and dasatinib was extended by 25%, which was superior to that of the single-agent group (quercetin 15%, dasatinib 10%) [118]. In addition to quercetin, goldfinch isoflavin, a soy isoflavone, has been shown to attenuate aging by modulating mitochondrial autophagy. Ovariectomized rats treated with goldfinch isoflavin showed activated mitochondrial autophagy and cleared abnormally functioning mitochondria, resulting in reduced ROS accumulation and DNA damage, as well as the downregulation of aging-associated secretory phenotypes [119]. Psoralen was found to activate mitochondrial autophagy by inhibiting the mTOR1 signaling pathway and significantly reducing fasting blood glucose, total cholesterol, low-density lipoprotein, and triglyceride levels in an aged mouse model. Improvements in these metabolic indexes were correlated with the enhancement of mitochondrial function, suggesting that it may slow aging by optimizing mitochondrial function [120]. The results showed that the lifespan of yeast, worms, and *Drosophila* was extended by treatment with the flavonoid 4,4′-dimethoxychalcone (DMC). DMC also slowed the aging process in human cell cultures [121]. Further studies revealed that DMC acts through GATA transcription factors to enhance autophagic flow and reduce p62 homologs in *Drosophila* brains [122]. The cGAS-STING pathway plays an important role in the pathogenesis of cellular inflammation and senescence diseases, making it an attractive drug target. Notably, STING signaling was aberrantly activated in senescent mouse model tissues, but the phosphorylation levels of STING and its downstream molecules were significantly reduced by ginkgetin treatment [123]. The results indicate that ginkgetin delays aging by specifically targeting STING, providing a new theoretical basis and potential drug candidate for anti-aging intervention.

#### Progress in clinical research and exploration of traditional Chinese medicine

In the current anti-aging research targeting mitochondrial autophagy, several clinical trials are actively exploring its translational potential. For example, UA, as a natural polyphenolic metabolite, has demonstrated its safety in human trials and has shown the potential to enhance mitochondrial gene expression and improve muscle function [124]. In addition, metformin is one of the most promising experimental anti-aging interventions at present, and clinical trials have shown that diabetic patients treated with metformin have a mild decline in cognitive function [125], but another trial showed that opposite result [126]. Overall, metformin is a very promising drug to slow down aging and prolong healthy life, with controllable side effects. NR is a NAD^+^ precursor substance, which shows good safety and tolerability during the treatment of PD patients, and provides a basis for the treatment of aging-related neurodegenerative diseases [127]. One of the pathways most obviously related to aging is the mTOR pathway, and clinical studies have found that the mTOR inhibitor RAD001 can improve the age-related decline of immune function in the elderly, confirming the scientific feasibility of targeting the mTOR pathway to improve immune function in the elderly [128, 129]. Dasatinib usually used in combination with the flavonol quercetin as senolytics, and relevant clinical trials have officially confirmed that this regimen has a systemic anti-aging effect, specifically inducing apoptosis of senescent cells, and simultaneously affecting aging markers in multiple tissues such as adipose tissue, skin, and circulatory system [130, 131]. Aging is a long-term change process accompanied by the occurrence of a variety of diseases. The western medicine used for anti-aging has long medication time and obvious side effects. Although traditional Chinese medicine has diverse ingredients and complex mechanisms, it has unique advantages in treating such diseases, which can meet medication needs and change clinical decisions. Astragali Radix has been proven effective in treating heart failure and activate mitochondrial autophagy to delay aging [132, 133]. Ginseng is believed to prolong life. Studies have confirmed that ginseng and its active ingredients can exert systemic anti-aging effects by regulating various aging-related pathways [134, 135]. Although several drugs have been proven to have good clinical efficacy, technological innovation and breakthroughs are still needed to ensure higher levels of safety, efficacy, and quality control for the high-quality development of Chinese medicines [136].

### Gene and biological therapy

Genetic engineering is the science of artificially modifying the genome of an organism using molecular biology techniques to add new characteristics or to treat disease by cutting, splicing, inserting, or deleting DNA. The use of genetic engineering techniques for the genetic and epigenetic control of cells has brought very favorable information to biotechnology and medicine [137]. Genetic engineering is related to mitochondrial autophagy and anti-aging research, which can slow the aging process by precisely regulating the autophagy pathway to enhance the ability of cells to “self-clean.”

#### CRISPR-Cas9

CRISPR-Cas9 is derived from the adaptive immune system of bacteria and archaea as a defense against exogenous genetic material. Its core components include a CRISPR array of direct repeats and spacers, and the Cas gene, which has nuclease activity to encode the Cas protein. By designing guide RNAs to pair complementarily with the target DNA, Cas9 generates double-strand breaks at specific locations. The cell subsequently achieves knockouts or insertions via non-homologous end-joining or homologous recombination repair [137, 138]. For example, the main pathological features of human PD must be simulated in PD research, but cannot be simulated by mouse and pig models. However, a non-human primate model with Parkin gene deletion can be successfully constructed using CRISPR-Cas9 technology, which not only solves the modeling challenge but also confirms the critical role of Parkin phosphorylation defects in the pathogenesis of PD [139]. Protein Kinase B (AKT) can directly regulate mitochondrial biosynthesis and function, which is conducive to improving mitochondrial function [140]. After the target editing of miR-29b using CRISPR-Cas9 in mice, the protein levels of its downstream target genes, insulin-like growth factor 1 and phosphatidylinositol 3-kinase, were significantly restored, which are the upstream activators of the AKT pathway.

#### TALEN

**Fig. 8:**
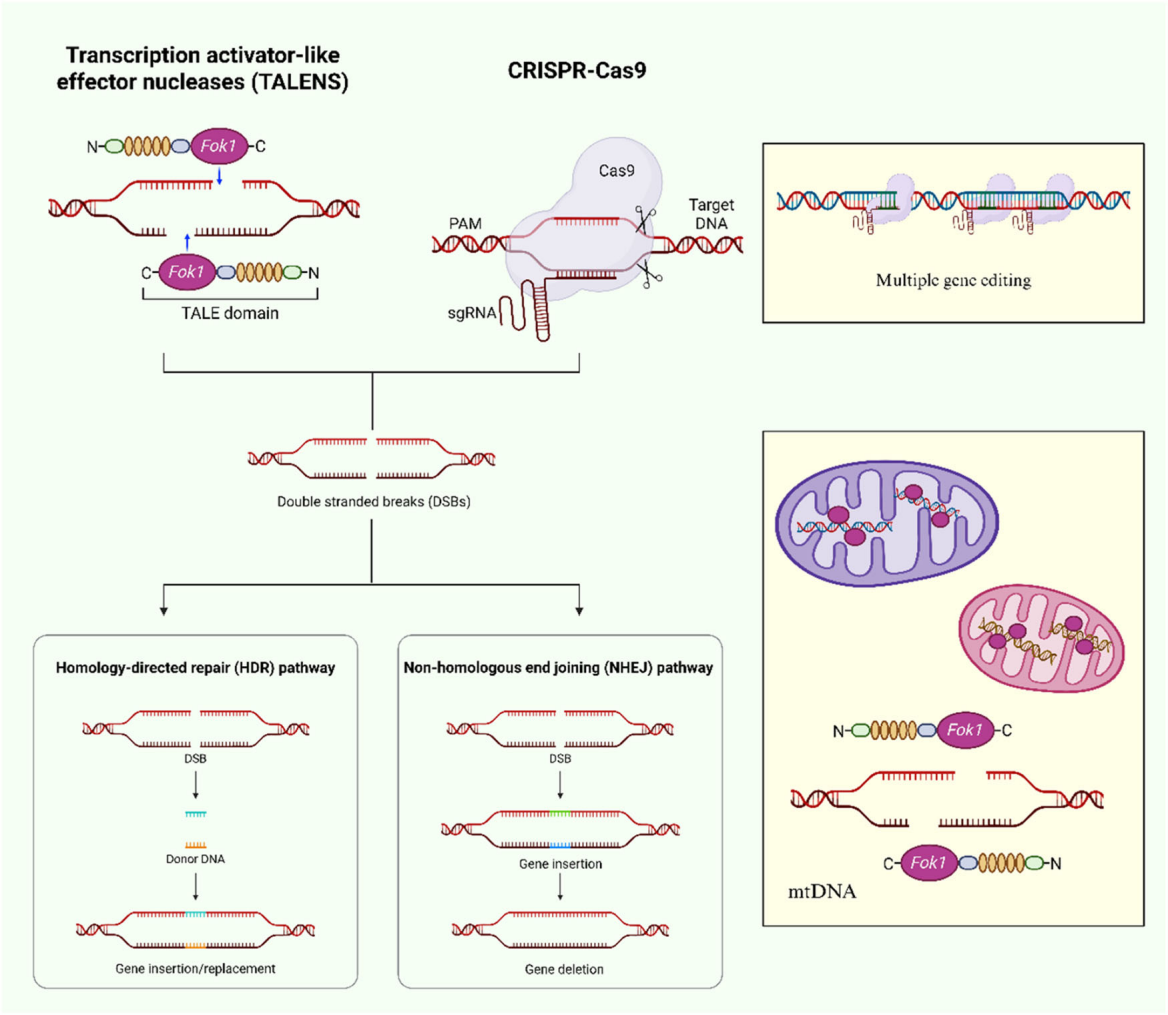
Comparison of TALEN with CRISPR-Cas9.

In addition to using CRISPR-Cas9 technology, TALEN is the choice in scenarios lacking suitable targets and needing extremely high specificity (Fig. 8). TALEN is a gene editing tool that is on par with CRISPR-Cas9. However, unlike CRISPR-Cas9’s recognition mode, TALEN relies on a protein-DNA recognition mode, which consists of two fusion proteins and thus has a higher molecular weight. mitoTALEN, which is derived from TALEN to target mitochondrial DNA, has been used more frequently, both for the specific recognition and cleavage of mtDNA sequences carrying disease-causing mutations and for the preferential removal of mutant mtDNAs in heterogeneous cells containing a mixture of wild-type and mutant mtDNAs. MitoTALEN has been shown to effectively target two common mtDNA mutations that both accumulate during aging and lead to mitochondrial dysfunction. Moreover, the treated heterogeneous cells were able to restore normal respiratory function and significantly enhance their oxidative phosphorylase activity [141]. Further studies found that the MitoTALEN system significantly reduced the proportion of mutant mtDNA in a treated mouse model of heterogeneous mitochondrial DNA harboring the m.5024 C > T mutation by specifically cleaving the m.5024 C > T mutation in the *tRNAAla* gene, which provides direct evidence for gene editing treatment of mitochondrial heter [142]. Thus, MitoTALEN shows great potential as a novel anti-aging intervention.

### Other modulations

A healthy life cannot be separated from good living habits, and diet and regular exercise in daily life are important signs of health. A reasonable diet includes the quantity, quality, and composition of food, as well as mealtime. Dietary regulation is more easily accepted by the public compared with drug treatment, and regulating nutritional availability will directly affect our health and life expectancy. Modifications include dietary interventions based on traditional eating habits, such as fasting, calorie restriction, the Mediterranean diet, and protein restriction, as well as newer dietary interventions, such as the ketogenic diet, glucose and carbohydrate restriction, amino acid interventions, and micronutrient interventions [143]. Caloric restriction was found to be effective in increasing the rate of mitochondrial protein synthesis in older individuals, primarily by activating the AMPK/SIRT1 signaling pathway, which improves the coordinated expression of nuclear and mitochondrial-encoded proteins [144]. Further studies have shown that caloric restriction significantly promoted mitochondrial biogenesis and improved mitochondrial electron transport chain activity, especially in the skeletal muscle and liver tissues of aging mice [145]. In addition, the ketogenic diet has been a hot topic in recent years, and researchers have found that the ketogenic diet not only promotes mitochondrial autophagy and protein renewal by inhibiting mTORC1 (35–50% reduction in activity) and the activation of AMPK (60–80% increase in activity), but also significantly enhances fat β-oxidation capacity by 40–60%, optimizes tricarboxylic acid cycle fluxes, and improves the aging-associated decline in mitochondrial metabolic efficiency [146].

Exercise conditioning is also important for healthy functioning and treating diseases, and exercise can significantly promote mitochondrial biogenesis to slow aging and ameliorate aging-related metabolic diseases [147]. Studies have shown that exercise training can restore the aging-induced decline in FUNDC1 protein levels, restore mitochondrial autophagy activity, and significantly delay the aging process of coronary artery endothelial cells in aged mice [148]. In addition, exercise was reported to upregulate PGC-1α expression, promote the dynamic balance between mitochondrial biogenesis and autophagy, and activate anti-inflammatory responses and neuroendocrine regulation [149]. These findings suggest that exercise can modulate mitochondrial autophagy in a multi-system and multi-targeted manner, and is one of the most promising non-pharmacological intervention strategies to slow aging.

## Summary and prospects

This paper systematically elaborates the central position of mitochondrial dysfunction in aging and its involvement in regulating mitochondrial autophagy. Mitochondrial autophagy is a key anti-aging target, and removing damaged mitochondria blocks ROS accumulation, mtDNA leakage, and inflammatory cascades (inhibition of the cGAS-STING/NLRP3 pathway). Autophagy activators significantly extend model organism lifespan by enhancing the PINK1/Parkin or receptor pathway. For example, UA upregulates mitochondrial gene expression to promote autophagy, NMN repairs metabolic networks via SIRT3 deacetylation, and metformin activates the AMPK-ULK1 axis. In addition, gene editing and lifestyle can likewise restore mitochondrial quality control and optimize autophagy. However, the shortcomings of the study are that, on the one hand, clinical data on most drugs (UA and NMN) are limited to short-term trials, and the long-term safety and tissue-specific effects are unknown. On the other hand, the model of aging research is limited, and experiments are mostly dependent on nematode/mouse models, which have significant physiological differences from humans. The complexity of the effects of gender and genetic background on autophagy regulation has also not been sufficiently explored. Therefore, human safety studies of drugs such as UA and NMN should be advanced to assess long-term anti-aging benefits. Meanwhile, combining AI models to predict autophagy dynamic equilibrium nodes to guide personalized anti-aging interventions should be performed. In summary, mitochondrial autophagy provides a revolutionary perspective for anti-aging; however, its complexity requires cross-scale studies and interdisciplinary collaboration. In the future, a balance between the depth of mechanism and clinical feasibility should be pursued, ultimately realizing the precise removal of damage and the remodeling of metabolic youth.
